# Impact of PNPLA3 I148M on Clinical Outcomes in Patients With MASLD

**DOI:** 10.1111/liv.16133

**Published:** 2024-10-16

**Authors:** Salvatore Petta, Angelo Armandi, Elisabetta Bugianesi

**Affiliations:** ^1^ Sezione di Gastroenterologia, Di.Bi.M.I.S University of Palermo Palermo Italy; ^2^ Division of Gastroenterology and Hepatology, Department of Medical Sciences University of Turin Turin Italy

**Keywords:** cardiovascular disease, hepatocellular carcinoma, liver fibrosis, metabolic dysfunction‐associated Steatotic liver disease, metabolic syndrome, PNPLA3, portal hypertension, single nucleotide polymorphisms

## Abstract

**Background and Aims:**

Metabolic dysfunction‐associated steatotic liver disease (MASLD) is a heterogenous clinical and histopathological entity, where multiple metabolic co‐factors are intertwined with high interindividual variability. The impact and severity of each factor (including obesity and type 2 diabetes) define a systemic dysmetabolism that can lead to either advanced liver disease and its complication (including hepatocellular carcinoma and clinical events related to portal hypertension) or extrahepatic events: incident cardiovascular disease, chronic kidney disease and extrahepatic cancers. The balance between environmental factors and genetic susceptibility has unique implications in MASLD: the intermittent injury of metabolic co‐factors, their fluctuation over time and their specific management, are counterbalanced by the presence of gene variants that can significantly impact the disease at multiple levels. The I148M variant in the PNPLA3 gene is the most investigated genetic susceptibility that induces a more severe steatohepatitis, enhanced fibrogenesis and can shape the incidence of long‐term clinical events regardless of, or worsened by, other metabolic risk factors.

**Methods and Results:**

In this review, we will summarise the updated evidence on the natural history of MASLD accounting for classical metabolic risk factors, the role of PNPLA3 in clinical sub‐phenotyping (e.g., ‘lean MASLD’), impact on disease severity and fibrosis progression, as well as its role for prognostication, alone or in combination with non‐invasive tools into polygenic risk scores.

**Summary**

- Metabolic dysfunction‐associated steatotic liver disease (MASLD) is a heterogeneous disease, with multiple metabolic co‐factors balanced with individual gene susceptibility.
- The natural history of MASLD is highly unpredictable, leading to hepatic as well as extrahepatic events, where both environmental and gene factors play a crucial role.
- I148M PNPLA3 is associated with MASLD disease severity and fibrosis progression even in the absence of classical metabolic dysfunction (including overweight or obesity).
- I148M PNPLA3 may be a valuable supporting tool in prognostication about mortality and liver‐related events including hepatocellular carcinoma.

**Abbreviations**
CAD: coronary artery disease
CVD: cardiovascular disease
HCC: hepatocellular carcinoma
IHD: ischemic heart disease
LSM: liver stiffness measurement
MACE: major adverse cardiovascular events
MASH: metabolic dysfunction‐associated steatohepatitis
MASLD: metabolic dysfunction‐associated steatotic liver disease
MBOAT7: membrane‐bound O‐acyltransferase 7
NHANES: National Health and Nutrition Examination Survey
PNPLA3: patatin‐like phospholipase domain‐containing protein 3
PRS: polygenic risk scores
SNPs: single nucleotide polymorphisms
T2DM: type 2 diabetes mellitus
TM6SF2: transmembrane 6 superfamily member 2
VCTE: vibration‐controlled transient elastography

## 1 Natural History of MASLD

Metabolic dysfunction‐associated steatotic liver disease (MASLD) is the most common form of chronic liver injury, with a prevalence of about 30% across countries [1]. The recent introduction of the umbrella term MASLD in place of the previous nomenclature Non‐Alcoholic Fatty Liver Disease highlights the connection of this steatotic liver disease with metabolic derangements [2]. Prevalence of MASLD has risen over the last decades due to the parallel increase of obesity and type 2 diabetes mellitus (T2DM), which are the main components of the metabolic syndrome and the most frequent clusters associated with MASLD. Indeed, MASLD prevalence rises to 75% in obese individuals [3] and 55% in patients with T2DM [4].

As a spectrum, MASLD defines a variety of liver injuries with different degrees of severity and progressiveness. Fat accumulation in the hepatocytes exceeding 5% defines the early stage of the disease, a relatively benign condition that can evolve toward superimposed inflammation and necroptosis of hepatocytes. Metabolic dysfunction‐associated steatohepatitis (MASH) arises from the perpetuation of mitochondrial oxidative stress, incomplete fatty acid oxidation with excessive harmful intermediates (e.g., ceramides, diacylglycerol), and endoplasmic reticulum stress under the constant metabolic cell stress driven by hepatic insulin resistance [5, 6, 7].

The recent advances in the understanding of metabolic pathways and crosstalk linked to insulin resistance and metabolic inflammation have shown a substantial intra‐ and interindividual MASH variability. Different subsets of metabolic dysfunctions may be present in the same individual (dyslipidaemia, arterial hypertension, T2DM, obesity) at different stages. Accordingly, different approaches may be undertaken by the subject, leading to a different degree of control of metabolic co‐factors. Otherwise then chronic viral or autoimmune hepatitis, which are paradigms of a persistent liver injury over time, metabolic dysfunction is intermittent and highly dependent on environmental as well as genetic susceptibility. In this context, single nucleotide polymorphisms (SNPs) in the PNPLA3 (patatin‐like phospholipase domain‐containing protein 3) gene has been associated with a worse phenotype, promoting the progression of MASLD in the absence of strong metabolic dysfunction (e.g., the ‘lean MASLD’ phenotype) [8, 9, 10].

These aspects are translated into a different degree of MASH activity (namely, the prevalence of ballooning and lobular inflammation) and different activation of Kuppfer cells and hepatic stellate cells. Liver fibrogenesis is not a uniform process, but rather an intermittent, yet progressive process that slowly leads to excessive scarring into the liver parenchyma toward advanced fibrosis stages and ultimately cirrhosis [11]. In this context, MASLD is probably the most unpredictable liver disease accounting for a high variability in the severity and progression of liver damage. However, if untreated, fibrosing MASH leads to cirrhosis and its complications, including hepatocellular carcinoma (HCC) and portal hypertension with a risk of clinical events (mainly ascites, variceal haemorrhage, hepatic encephalopathy) potentially requiring liver transplantation [12, 13]. This is particularly relevant for high‐risk groups, including obese and diabetic patients, where the prevalence of MASH and advanced fibrosis are superior to the general adult population: 33.7% MASH and 21% significant fibrosis in the obese individuals [3]; 37% MASH and 17% advanced fibrosis in diabetic individuals [4].

If advanced fibrosis bears the risk for liver‐related events and all‐cause mortality [13, 14], the complex picture of metabolic syndrome is responsible for the clinical burden in patients with low fibrosis stages, but across all spectrum of MASLD, including cirrhosis. Cardiovascular disease (CVD) is the major driver of prognosis in patients with MASLD without advanced fibrosis, followed by extrahepatic cancers and chronic kidney disease [15]. However, a prospective multicentric study showed that over a median of 5.5 years, CVD incidence remains higher than liver‐related events even in bridging fibrosis (F3) [16]. In one Swedish population‐based study, MASLD patients had a higher risk of developing major adverse cardiovascular events (MACE), with highest incidence observed in cirrhotic (F4) patients [17]. Overall, a patient with MASLD is at risk for developing either hepatic or extrahepatic events, with metabolic risk factors driving fibrogenesis and clinical outcomes. In this view, one of the current efforts is to establish comprehensive models of care that consider all metabolic co‐factors, a hierarchy across all factors and the intersection with genetic background. The objective of future models of care would be the identification of ‘which’ MASLD in ‘which’ patient, a paradigm of precision medicine that is highly required in this field [18].

## 2 Metabolic Risk Factors as a Drivers of Clinical Outcomes in MASLD

If we consider all features of metabolic syndrome including MASLD, then the current clinical and economic disease burden results are relevant, but most importantly if we consider the future trajectories, then an enormous burden for healthcare systems is depicted [19]. In European countries, even if obesity and T2DM are controlled over the next years, MASH prevalence will increase 15%–56%, while liver‐related mortality and advanced liver disease will be more than doubled [20]. Incidences of decompensated cirrhosis and HCC due to MASLD are projected to increase by 168% and 137% by 2030, with an estimated 800.000 excess liver‐related deaths from 2015 to 2030. In one analysis involving more than 8 million individuals from 22 countries, HCC incidence among MASLD was 0.44 per 1000 person‐years, while liver‐related and overall mortality in MASH individuals were 11.7 and 25.5 per 1000 person‐years [21]. Recent phenome‐wide association analysis and temporal disease trajectory analysis conducted on 163.303 MASLD individuals (UK Biobank) provided evidence of the interconnection of multiple metabolic co‐factors in MASLD. Over 13 years, MASLD patients developed 113 medical conditions and 8 causes of death, through intermediate conditions including T2DM, hypertension, obesity, CVD and smoking habit (with acute myocardial infarction as the primary leading cause of disease). Malignant neoplasms and CVD were the main causes of death [22].

Among metabolic risk factors, T2DM is the most impacting on the progression of MASLD. In a prospective study of 524 US diabetic patients, 14% and 6% resulted as having advanced fibrosis and cirrhosis, respectively [23]. In one updated meta‐analysis of 11 231 individuals from the National Health and Nutrition Examination Survey (NHANES) III (follow‐up to 2019), MASLD individuals (7.8%) had the highest risk for age‐related mortality (32.7%) as compared to non‐MASLD, after a median follow‐up of 26.7 years. Among MASLD subjects, the highest age‐standardised cumulative mortality was observed in those with T2DM (41.3%) (pairwise *p* < 0.04). Individuals bearing both MASLD and T2DM reached the highest hazards for all‐cause and CVD‐related death (HR 4.7 and 20.01, respectively) [24]. A recent Korean nationwide population‐based study of almost 8 million participants, showed that 5‐year incident rates of CVD display a 6‐fold increase in those with severe steatotic liver disease defined by Fatty Liver Index and with T2DM, as compared to those without T2DM (3.7‐fold increase) [25]. Another study remarked on the relevance of the duration of T2DM, showing a doubled risk to develop CVD in MASLD patients with a 5‐year standing T2DM [26]. In one individual participant‐level data meta‐analysis of 2016 MASLD patients from six cohorts (USA, Japan, Turkey), 105 patients developed liver decompensation, of which those with T2DM resulted in the greatest 1‐ to 5‐year risk (from 3.37% to 13.8% versus 1.07% to 3.9% in non‐T2DM individuals, *p* < 0.0001). Additionally, T2DM was independent predictor of liver decompensation (aHR 2.1, *p* = 0.0006 and HCC 5.3, *p* = 0.004) [27].

The role of metabolic co‐factors in the cumulative risk of outcomes underlines the holistic approach that would be advisable in this population. A retrospective multicentric Italian study showed that a major proportion of overweight or obese MASLD patients had hypertension, T2DM and increased carotid intima‐media thickness, as compared to lean MASLD [28]. Another multicentric cross‐sectional prospective study of about 1000 biopsied MASLD patients showed that the number of metabolic co‐factors determined the risk for MASH and significant fibrosis (F2‐F4), in both obese (OR 3.47 and 3.89, respectively) and non‐obese (OR 3.70 and 3.92, respectively) [29]. A recent meta‐analysis of 129 studies showed that MASLD individuals, compared to non‐MASLD, displayed higher hazards for cardiovascular events (HR 1.43, *p* < 0.01), across incident metabolic events: arterial hypertension (HR 1.75), T2DM (HR 2.56), chronic kidney disease (HR 1.38) [30]. In particular, a machine learning‐based approach on nearly 3000 MASLD patients showed that over 7 years, individuals aged > 60 years and with at least 4 metabolic risk factors had a higher risk of developing MACE, suggesting a way for the identification of high‐risk MASLD sub‐populations [31]. This is confirmed by a previously mentioned study from the NHANES cohort, where also the cluster ‘metabolically unhealthy’ (namely, any component of metabolic syndrome without T2DM or pre‐T2DM) displayed increased mortality among MASLD individuals, accounting for 30% of the total [25].

A metabolic connection between organs may be represented by microvascular damage, which can be driven by insulin resistance, arterial hypertension or atherogenic dyslipidaemia. A Swedish nationwide population‐based cohort study showed a higher, independent risk for MASLD individuals to develop microvascular damage (composite endpoint including chronic kidney disease, retinopathy, neuropathy) over 5.7 years, with HR 1.45 adjusted for hypertension, dyslipidaemia and time‐varying T2DM [32]. To corroborate these findings, data extrapolated from the Framingham Study revealed that individuals with MASLD have smaller total cerebral brain volume after multiple adjustments and in the absence of known cerebral disease [33].

The endothelial dysfunction is also relevant if applied to macrovascular damage driven by atherosclerosis [15]. A recent meta‐analysis of 24 observational studies reported positive odds for developing critical coronary stenosis and ‘high‐risk’ features in coronary plaques by computed tomography coronary angiography (OR 1.54 and 2.13, respectively) [34]. The dynamicity and intermittence of metabolic co‐factors have an impact on incident outcomes, especially when considering cardiovascular events. One prospective study of 10 thousand individuals from the Framingham Study, the Coronary Artery Risk Development in Young Adults Study and the Multi‐ethnic Study of Atherosclerosis, showed that the association between hepatic steatosis and incident CVD (with HR 1.21 adjusting for baseline variables) was attenuated when the model was adjusted for time‐varying variables. In particular, the association was not statistically significant if body mass index was analysed as time‐varying co‐variate [35]. As a consequence, the risk of incident CVD was reduced by 49% in MASLD individuals undergoing bariatric surgery for morbid obesity [36].

**FIGURE 1:**
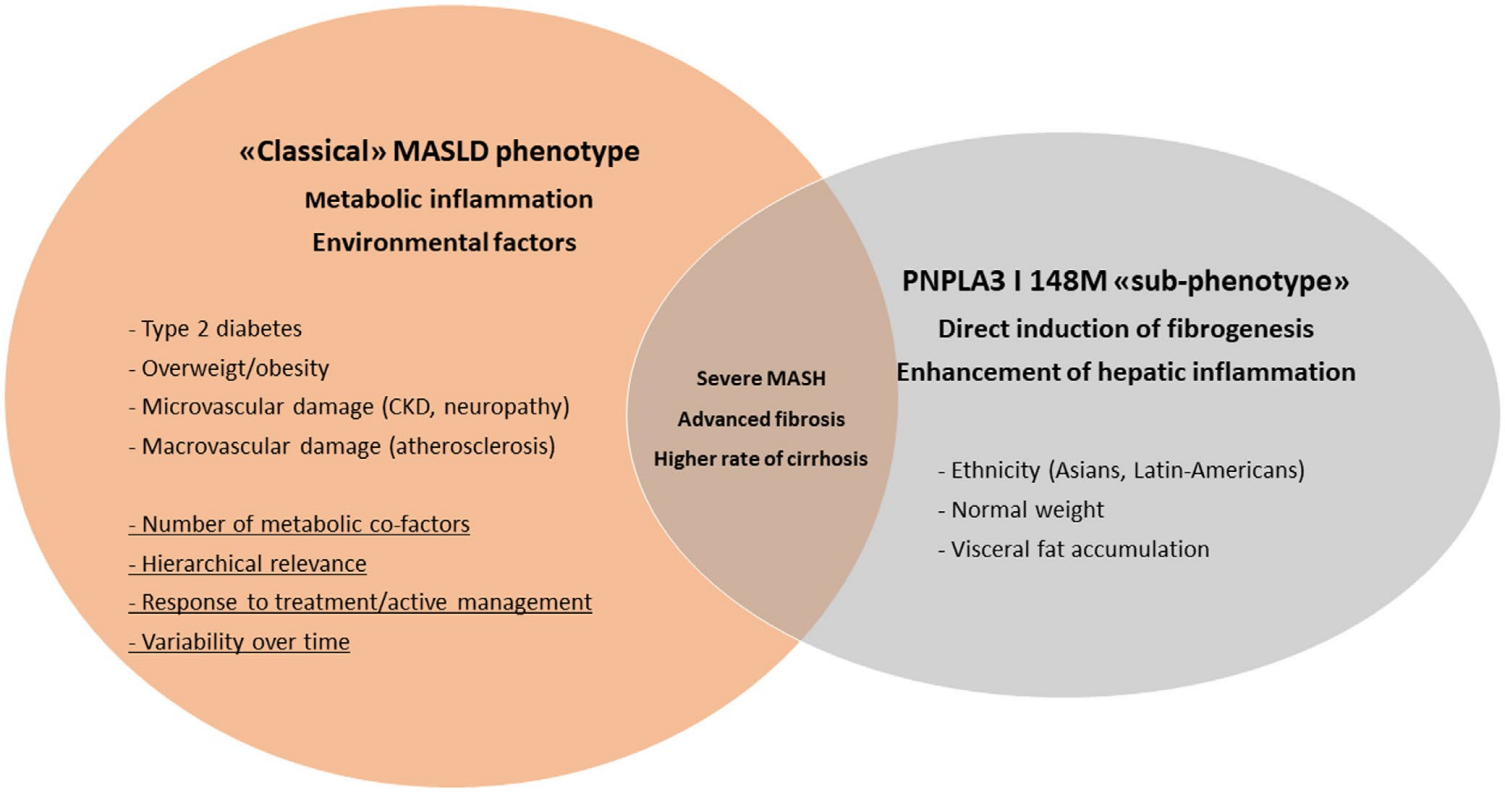
Clusters of metabolic dysfunction‐associated steatotic liver disease (MASLD) phenotype: the impact of environmental factors and the PNPLA3 I148M variant sub‐phenotype on the severity of liver disease. CKD, chronic kidney disease; MASH, metabolic dysfunction‐associated steatohepatitis.

Taken together, these data highlight the relative impact of a major metabolic co‐factor on a systemic metabolic dysfunction that drives prognosis in MASLD patients. The heterogeneity and dynamicity of these factors shape a unique phenotype at the individual level (Figure 1). However, the balance between environmental factors and gene susceptibility represents a further complexity in MASLD, being able to drive prognosis (hepatic and extrahepatic outcomes) as well as to define specific ‘sub‐phenotypes’ of disease severity that would demand special consideration.

## 3 PNPLA3 I148M and Severity of Liver Disease in MASLD

Beyond classical metabolic risk factors, ethnicity and gene background account for a relevant proportion of MASLD ‘sub‐phenotypes’. A meta‐analysis of 94 studies from 24 countries showed that globally 40% of patients with MASLD are classified as non‐obese, with 19.2% being lean. This population is burdened by similar long‐term liver‐related as well as non‐liver‐related mortality (4.1 and 4.0 per 1000 person‐years, respectively) [37]. The same trends are observed about CVD‐related mortality, described in another meta‐analysis of 110.000 MASLD patients [38]. Studies conducted on ethnical clusters suggest that Caucasian lean MASLD are predominantly male and with younger age, sharing the same prognosis of non‐lean counterparts (liver‐related events and all‐cause mortality) [39], and seem to be characterised by a more aggressive severe histological phenotype [40]. In the Asian populations, lean MASLD had evidence of poorer prognosis, with a higher incidence of acute coronary syndrome [41], as well as all‐cause mortality and extrahepatic cancers [42]. The disconnection between MASLD prognostication in the absence of excessive body weight, which is the cornerstone of classical disease onset, may be partially explained by genetic background. The rs738409 C > G variant is higher in Asian a Latin‐American individuals, as compared to Caucasian individuals [43]. An Asian population study of 904 individuals showed that lean patients were more likely to carry the *PNPLA3* rs738409 GG (homozygous) variant (30% of cases). Additionally, the GG phenotype was associated with the greatest increase in the MASLD risk in lean individuals (OR 6.04), as compared to overweight and obese [44]. The severity and distribution of visceral adipose tissue accumulation is a hallmark of early metabolic derangement impacting liver phenotype, potentially being shaped by gene background. In one Asian prospective study, visceral fat accumulation was significantly associated with significant liver fibrosis only among *PNPLA3* rs738409 C > G carriers (aOR 1.05 for GG homozygosity; aOR 1.03 for CG heterozygosity) [45].

The role of PNPLA3 in defining the MASLD phenotype has been investigated over the last decades. PNPLA3 gene encodes for adiponutrin, a key liver enzyme involved in the export of fats from hepatocytes. Polymorphisms in the PNPLA3 gene cause a reduced enzyme functionality, entrapping fats into the liver parenchyma. This is associated with enhanced steatosis and major susceptibility to superimposed MASH and fibrosis. A genome‐wide association study performed across the whole MASLD spectrum on 1483 European cases matched with controls, confirmed PNPLA3 as a risk factor for the full histological spectrum including fibrosis [46]. This robust evidence corroborated the early evidence on the impact of the I148M PNPLA3 variant in the MASLD disease activity, where it was initially associated with liver steatosis (*p* = 0.03), portal inflammation (*p* < 0.05), lobular inflammation (*p* = 0.005), Mallory‐Denk bodies (*p* = 0.015) and fibrosis (*p* < 0.05), with a slight but positive significance of younger age of diagnosis (*p* = 0.045) [47]. Similarly, early meta‐analysis supported the role of the PNPLA3 I148M variant as a strong modifier of MASLD, conferring a more aggressive disease [48, 49]. This meta‐analysis found that GG carriers had a 3.24‐fold greater risk of necroinflammatory scores and a 3.2‐fold greater risk of developing fibrosis, as compared to wild‐type counterparts. MASH was more associated with the G allele (OR 3.4), with a 28% increase in serum transaminases [50].

Gene analysis was performed in the aforementioned Italian multicentric study on biopsy‐proven MASLD individuals, where the *PNPLA3* rs738409 C > G variant was the only variable independently associated with lean MASH and significant fibrosis (F2 or greater) [28]. Similarly, a prospective Scandinavian study on 546 MASLD patients showed that *PNPLA3* GG homozygosity was associated with a higher prevalence of baseline MASH (OR 3.67) and that after a median follow‐up of 40 years, the same genetic profile was associated with a higher rate of severe liver disease (aHR 2.27 compared with the CC wild‐type phenotype). Additionally, PNPLA3 GG accentuated the rate of cirrhosis onset among MASLD patients (aHR 23.3) [51]. Finally, a recent causal mediation analysis was performed to understand at which histological level PNPLA3 mutation acts on promoting fibrosis development. In nearly half of cases, PNPLA3 seems to promote fibrosis directly through specific fibrogenesis pathways; in the remaining cases, the variant allele seems to act indirectly through the mediation of portal inflammation [52].

The growing understanding of the causal role of PNPLA3 I148M variant on liver disease severity has led to investigations on potential non‐invasive assessment of liver injury and fibrosis by gene‐including scores. In prediction models for the identification of liver fat content by magnetic resonance proton density fat fraction (MR‐PDFF), carriage of PNPLA3 risk allele was identified as causing 8.7% higher steatosis [53]. Similarly, *PNPLA3* rs738409 C > G variant reached significance among independent gene variants that are associated with intrahepatic disease activity evaluated by MR‐based corrected T1 [54]. Moreover, a combination of PNPLA3 I148M genotype with a surrogate marker of insulin resistance (namely, the enhanced lipoprotein insulin resistance index by magnetic resonance spectroscopy) could improve the identification of MASH (defined by a NAFLD Activity Score [NAS] ≥ 3) with an area under the curve (AUC) of 0.82 [55]. For the identification of severe MASH (NAS ≥ 5), another study found that the addition of PNPLA3 GG trait to a model including age, sex, BMI, T2DM and ALT, augmented the AUC from 0.75 to 0.77, while for the prediction of advanced fibrosis (F3–F4), adding PNPLA3 GG trait to the baseline model yielded to AUC 0.78 [56].

Finally, the formulation and validation of polygenic risk scores (PRS) are among the most promising tool for the non‐invasive identification of MASH and fibrosis. In one prospective study of diabetic individuals, a PRS including PNPLA3, transmembrane 6 superfamily member 2 (TM6SF2) and SERPINA1 was associated with an increased risk of cirrhosis (*p* = 0.03) and, more interestingly, was associated with advanced fibrosis among those with FIB‐4 < 1.3 (*p* = 0.03) [57]. In addition, the inclusion of a PRS‐HFC (hepatic fat content) including PNPLA3, TM6SF2, membrane‐bound O‐acyltransferase 7 (MBOAT7) and GCKR in more than 250.000 individuals in the UK Biobank, revealed enhanced stratification of subjects in intermediate and high‐risk classes of fibrosis scores, in addition to metabolic risk factors [58].

These data further support the use of gene‐based non‐invasive tools for risk stratification across the MASLD spectrum, giving a strong contribution in deep patient phenotyping.

## 4 PNPLA3 I148M and Risk of Fibrosis Progression in MASLD

In patients with MASLD, the severity of liver fibrosis evaluated by histology or by non‐invasive scores is the main driver of complications and prognosis. An aggregated data meta‐analysis reported that the risk of developing liver‐related events in MASLD patients with F2, F3 or F4 fibrosis is 2.6, 5.2 and 12.7 times higher, respectively, when compared to MASLD patients without liver fibrosis [59]. Consistent with these data, in a large multicentre cohort of patients with histological or clinical diagnosis of MASLD it was demonstrated that in patients with F0‐F1 fibrosis by histology or in those at low risk of advanced fibrosis by vibration‐controlled transient elastography (VCTE) (liver stiffness measurement [LSM] < 8 KPa) the risk of liver‐related events was negligible, while it was clinically significant in patients with F2 fibrosis or at intermediate risk of advanced fibrosis (LSM > =8 − < 9.6), and high in those with F3‐F4 fibrosis or at high risk of advanced fibrosis (LSM > =9.6 KPa) [60]. Notably, in these cohorts it was also observed a significant risk of extrahepatic complications progressively increasing according to the severity of liver fibrosis [60].

Nevertheless, the clinical relevance of fibrosis, the rate of fibrosis progression over time is not linear and it is possible to observe patients with indolent liver disease and with very slow/absent progression of fibrosis over time, and on the other hand, the so called ‘rapid progressor’ able to develop cirrhosis in few years [61]. Different factors have been involved in this heterogeneity like components of metabolic syndrome—especially obesity, T2DM and arterial hypertension, menopausal status in females, ageing, etc. In this complex landscape, genetic background and in particular the presence of the *PNPLA3* rs738409 C > G variant could account for some variability. Consistently, in an Italian cohort of 430 and 342 MASLD patients with availability of FIB‐4 and LSM, respectively, at baseline and last follow‐up visit, and with a median follow‐up of 54 months, it was demonstrated that the PNPLA3 genotype well stratified the risk of fibrosis progression [62]. Specifically, when using FIB‐4, fibrosis progression was observed in 16%, 19% and 32.8% of patients with PNPLA3 CC/CG/GG genotype, with an adjusted odds ratio of 1.65. Similarly, when using LSM, the progression of liver fibrosis was reported in 8.1%, 13.2% and 23.2% of patients with PNPLA3 CC, CG and GG genotypes, respectively, with an adjusted odds ratio of 1.90 [62]. Along this line, in two large studies on patients with steatosis defined by increased ALT serum levels, from the Michigan Genomics Initiative—7893 individuals—and from the UK Biobank—46 880 individuals—*PNPLA3*‐rs738409‐GG genotype was associated with higher incidence rate of cirrhosis in both cohorts [63]. Notably, the Authors found a positive interaction between the *PNPLA3*‐rs738409‐GG and metabolic disorders like T2DM and obesity [63]. Similar results were reported in a meta‐analysis of two large population cohort studies—110 761 individuals from Copenhagen and 334 691 individuals from the UK Biobank—where the authors applied a genetic risk score for steatosis ranging from 0 to 6 and also included *PNPLA3* rs738409 C > G variant, to predict the development of cirrhosis [64]. Individuals with scores 1, 2, 3, 4 and 5 or 6 had odds ratios for cirrhosis of 1.6, 2.0, 3.1, 5 and 12, when compared with those with a score of 0 [64]. The clinical meaning of all these results is further enriched by the synergistic interaction existing between the PNPLA3 genotype and both alcohol intake and obesity. In a large UK Biobank cohort of more than 400 000 participants, 2398 of them developed cirrhosis, and the adjusted risk of cirrhosis was 17 times higher in individuals with excessive drinking, with obesity, and with PNPLA3 GG genotype, when compared to those without any of these 3 risk factors [65]. All the above‐reported evidence could support the use of genetic testing in clinical practice for stratifying the risk of liver fibrosis progression and cirrhosis development in at‐risk patients.

## 5 PNPLA3 I148M and Risk of Liver‐Related Events in MASLD

The research agenda in patients with MASLD is not only focused on the identification of patients with significant or advanced liver fibrosis, but also on the ability to non‐invasively predict the risk of developing liver‐related events and liver‐related mortality. For this purpose, different studies and an individual patient data meta‐analysis demonstrated that non‐invasive tests/tools elaborated and used for fibrosis diagnosis—such as FIB‐4, LSM, AGILE3+ score, MRE, etc—well predict the prognosis of MASLD patients and—most relevant—have similar performance or sometimes outperform the accuracy of liver fibrosis assessed by the gold standard histology [66, 67, 68, 69]. In this scenario, the identification of unconventional risk factors for poor prognosis, such as genetic background, can add insights in the pathophysiology of MASLD opening also new frontiers toward non‐invasive prediction.

Both cross‐sectional and longitudinal studies highlighted an association between the *PNPLA3* rs738409 C > G variant and the risk of liver‐related complications in patients with MASLD.

A longitudinal Asiatic study on a cohort of 1550 patients with histological diagnosis of MASLD, reported that the 5‐year rate of liver‐related events (HCC or hepatic decompensation) progressively increased from 0.5% in patients with PNPLA3 CC genotype, to 3.8% and further to 5.8% in those with CG or GG genotype, respectively, with an adjusted HR of about 16 for patients carrying the at‐risk *PNPLA3* rs738409 C > G variant [70]. Similarly, a study on patients with MASLD and diabetes found that the PNPLA3 GG genotype independently predicted a higher risk of liver complications (HCC and oesophageal varices) [71]. Some other studies searched for identifying clusters of patients where the effect of the PNPLA3 variant is more pronounced. Accordingly, a recent multicentre international collaborative study found that MASLD non‐obese female patients older than 50 years, if carrying the PNPLA3 GG risk genotype, are at higher risk of developing liver‐related events compared with their counterpart with the CC and CG genotype [72]. When looking separately at liver decompensation and HCC further data are available. Grimaudo and colleagues followed 471 consecutive patients with histological diagnosis of MASLD or with clinical diagnosis of compensated MASLD‐related cirrhosis observing that in a median follow‐up time of 64 months the *PNPLA3* rs738409 C > G variant was independently associated with a higher risk of developing liver decompensation both in the entire cohort and the subgroup of patients with F3 liver fibrosis or cirrhosis [73]. Notably, the association was confirmed also after adjusting for the severity of baseline liver fibrosis [73]. Similarly, a Swedish study on patients with MASLD observed that the PNPLA3 GG genotype independently predicted the occurrence of both liver decompensation and HCC [51]. Along this line, in a small Asiatic cross‐sectional cohort of patients with MASLD‐related cirrhosis, Shao et al. also reported that the presence of the homozygous GG genotype of PNPLA3 was independently associated with a 3‐time increased risk of presence of clinical manifestations of hepatic decompensation [74]. Moving from liver decompensation to HCC, several data are now available. The before quoted longitudinal Italian study from Grimaudo and colleagues provided evidence that the *PNPLA3* rs738409 C > G variant is a risk factor for the occurrence of HCC, by increasing the risk of 2.5 times also after adjusting for clinical and metabolic risk factors as well as for baseline liver disease severity [73]. Along this line, a study comparing 100 European patients with MASLD‐related HCC to 275 patients with histological diagnosis of MASLD but without HCC highlighted a higher prevalence of the at‐risk PNPLA3 allele in the HCC group, also showing an adjusted 2‐time and 5‐time higher risk of HCC in patients with CG and GG genotype, respectively [75]. Noteworthy, the same study reported a more pronounced effect (2.5‐ and 12‐time higher risk in CG and GG genotypes) in the population‐based setting of UK Biobank [75]. The association between rs738409 C > G variant and the risk of HCC in MASLD has been also underlined in a meta‐analysis, where on subgroup analysis in MASLD‐related or alcohol‐related cirrhosis the PNPLA3 at risk variant increased the risk of HCC of 1.67 folds [76]. The effect of PNPLA3 on HCC risk, as previously reported for cirrhosis development, is also modulated by environmental factors like alcohol use and obesity. In 414 209 individuals from UK Biobank, followed for a median time of 10.9 years, there was a synergistic interaction between PNPLA3 genotype, alcohol intake and obesity; subjects with all three 3 factors had an adjusted HCC risks 30 times higher than their counterparts without all 3 risk factors [65]. Another key point interfering with the impact of the *PNPLA3* rs738409 C > G variant on HCC is the interaction with other genetic variants affecting HCC development. A genome‐wide association study on case–control derivation and validation cohorts, analysed separately and then pooled by meta‐analysis identified five regions associated with HCC, including *PNPLA3* rs738409; notably, the combination of homozygous variants of PNPLA3 and TERT revealed a 6.5‐fold higher risk for HCC respect to wild‐type subjects [77]. Along this line, the growing number of at‐risk alleles in PNPLA3 (rs738409), TM6SF2 (rs58542926) and MBOAT7 (rs641738) genes increased the risk of HCC [78]. This issue has been used to develop genetic or clinical‐genetic scores to stratify the risk of developing HCC. Bianco and colleagues applied the aforementioned PRS‐HFC by putting together common genetic variants (*PNPLA3* rs738409—TM6SF2 rs58542926 –GCKR rs1260326—MBOAT7 rs641738). The score stratified the risk of HCC in patients with and without cirrhosis and both patients with liver disease referred to hepatological centres and UK general population [79]. The same score has been also combined with NFS and FIB‐4 improving in the UKB population, their accuracy in the prediction of not only cirrhosis but also liver events in the entire population and subgroups stratified according to obesity, diabetes and steatosis [58]. Similarly, the GEMS (Genetic and Metabolic Staging) score based on the combination of clinical and metabolic parameters with common variants in PNPLA3, HSD17B13 and TM6SF2 genes predicted the occurrence of liver events in MASLD patients with FIB‐4 ≥ 1.3 and, at a lower accuracy, in UK Biobank population [80].

The demonstrated link between the *PNPLA3* rs738409 variant and the risk of developing both liver decompensation and HCC raises the suspicion that patients carrying this allele could also be at increased risk of mortality. Likewise, in this setting, we could discriminate between population‐ and patient‐based studies. A cohort study on 110 913 Danish individuals with a median follow‐up of 9.5 years, demonstrated that subjects homozygous for the *PNPLA3* rs738409 variants had a 3‐fold higher risk of liver‐related death when compared to those without any at‐risk allele, the risk further increasing in presence of other at‐risk alleles (TM6SF2 rs58542926 and HSD17B13 rs72613567) [81]. The evidence from this study confirmed what was previously reported by Unalp‐Arida and colleagues in the US population from the third US NAHNES. They observed that patients carrying one or two *PNPLA3* rs738409 G alleles had an adjusted risk of liver‐related mortality of 2.9 and 18.2 folds higher, respectively, compared to wild‐type individuals [82]. Along this line, data from the UK Biobank general population on more than 400 000 participants, as before showed for cirrhosis and liver‐related events, showed that PNPLA3 variant, obesity and alcohol use had a synergistic effect on the risk of liver‐related mortality, subjects with all the three risk factors having a 21 higher risk of liver‐related mortality respect to those without [65]. When moving from the general population to patients with MASLD an Italian single centre study also reported that in 471 MASLD patients followed for a median of 64.6 months, *PNPLA3* rs738409 C > G variant other than to increase the risk of liver decompensation and HCC, also exposed to a 3.5 times higher risk of liver‐related death, while no association was found with overall mortality [73].

Finally, available data demonstrate that the presence of 1 or 2 *PNPLA3* rs738409 C > G variants progressively increase the risk of developing liver‐related events and HCC and of experiencing liver‐related mortality. Notably, these associations are modulated by additional metabolic risk factors and their weight can change according to clinical settings from the general population to at high‐risk MASLD. Prospective studies are needed to assess the potential clinical use of genetic testing for stratifying prognosis and personalised follow‐up.

## 6 *PNPLA3* I148M and Risk of Extrahepatic Events in MASLD

**FIGURE 2:**
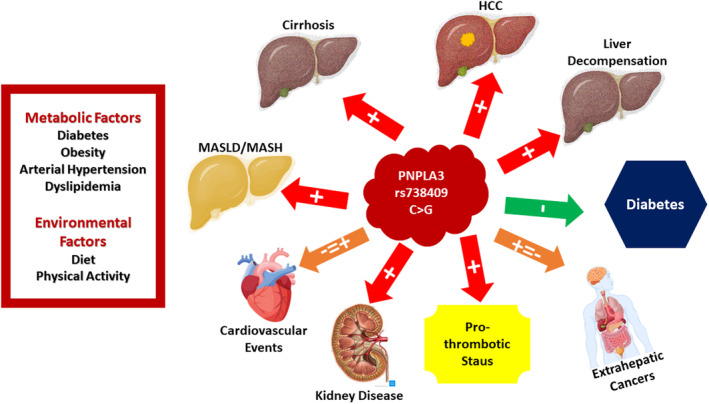
Association between PNPLA3 rs738409 C > G variant and both hepatic and extrahepatic complication. HCC, hepatocellular carcinoma; MASLD, metabolic dysfunction‐associated steatotic liver disease; MASH, metabolic dysfunction‐associated steatohepatitis.

MASLD is a systemic disease with a high burden of metabolic comorbidities and with a high rate of mortality related to extrahepatic cancers and cardiovascular events, the last being the first cause of death in this population [83]. Moreover, MASLD has been also associated with other extrahepatic disorders like Inflammatory Bowel Disease, psoriasis, etc. This landscape accounts for common pathophysiological mechanisms—mostly insulin resistance—surrounding both MASLD and extrahepatic disorders but also points to a direct role of MASLD and especially MASLD‐related hepatic and systemic inflammatory and profibrogenic status in increasing the risk of extrahepatic complications [83]. In this complex setting, it is plausible to ask whether genetic background, and especially the most validated genetic risk factor for MASLD and its severity, that is, *PNPLA3* rs738409 C > G variant, can also affect the risk of extrahepatic complications. Table 1 and Figure 2 resume available data about the association between the PNPLA3 C > G variant and extrahepatic complications in MASLD.

**TABLE 1:** PNPLA3 rs738409 variant and risk of developing liver‐related and extrahepatic outcomes in MASLD.

Reference	Region	Design	Setting	Outcome
Liver‐related outcomes				
Liu et al. [75]	Multicentre	Case–control	MASLD‐HCC and biopsy‐proven MASLD	Adjusted 2 time and 5‐time higher risk of HCC in patients with PNPLA3 CG and GG genotype
Liu et al. [75]	UK	Longitudinal	General population	2.5‐ and 12‐time higher risk of HCC in PNPLA3 CG and GG genotype
Singal et al. [76]	Multicentre	Meta‐analysis	Cirrhosis	PNPLA3 C > G at risk variant increased the risk of HCC of 1.67 folds
Grimaudo et al. [73]	Italy	Longitudinal	Biopsy‐proven MASLD or clinical diagnosis of compensated MASLD‐related cirrhosis	PNPLA3 C > G variant independently associated with a higher risk of decompensation, HCC and liver‐related death
Unalp‐Arida et al. [82]	US	Longitudinal	General population	PNPLA3 CG and GG had an adjusted risk of liver‐related mortality of 2.9 and 18.2 folds higher compared to wild‐type
Holmer et al. [51]	Sweden	Longitudinal	MASLD	PNPLA3 GG independently associated with a higher risk of decompensation and HCC
Shao et al. [74]	Japan	Longitudinal	Cirrhosis	PNPLA3 GG associated with a higher risk of hepatic decompensation
Gellert‐Kristensen et al. [81]	Danish	Longitudinal	General population	PNPLA3 GG associated with a 3‐fold higher risk of liver‐related death
Seko et al. [70]	Japan	Longitudinal	Biopsy‐proven MASLD	PNPLA3 CC/GG independently associated with a higher risk of liver‐related event
Rosso et al. [72]	Multicentre	Longitudinal	Biopsy‐proven MASLD	MASLD non‐obese female patients older > 50 years, if carrying the PNPLA3 GG risk genotype, are at higher risk of developing liver‐related events
Hassan et al. [77]	North America	Case–control	HCC and control	By genome‐wide association study PNPLA3 C > G variant increased HCC risk of 1.66 folds
Lavrado et al. [71]	Brazil	Longitudinal	MASLD and diabetes	PNPLA3 GG associated with cirrhosis complications
Cardiovascular outcomes				
Lauridsen et al. [84]	Multicentre	Genome‐wide association study	Case–control	PNPLA3 C > G protective against coronary artery disease
Meffert et al. [85]	Germany	Longitudinal	General population with/without MASLD	PNPLA3 C > G protective against cardiovascular‐related mortality only in males without steatosis
Lauridsen et al. [84]	Danish	Longitudinal‐Mendellian randomisation	General population	PNPLA3 C > G protective against ischemic heart disease
Wijarnpreecha et al. [86]	US	Longitudinal	General population with/without MASLD	PNPLA3 C > G confers a trend for an increased cardiovascular‐related death in the entire population but not in MASLD patients
Unalp‐Arida et al. [82]	US	Longitudinal	General population	PNPLA3 C > G not associated with cardiovascular‐related death
Akuta et al. [87]	Japan	Longitudinal	Biopsy‐proven MASLD	PNPLA3 C allele independently predicted cardiovascular events
Xia et al. [88]	China	Longitudinal	General population	PNPLA3 C > G protective against cardiovascular mortality
Holmer et al. [51]	Sweden	Longitudinal	MASLD	PNPLA3 C > G not associated with cardiovascular‐related death
Gellert‐Kristensen et al. [81]	Danish	Longitudinal	General population	PNPLA3 C > G not associated with cardiovascular‐related death
Semmler et al. [89]	Austria	Longitudinal	General population	PNPLA3 C > G not associated with cardiovascular‐related death
Tai et al. [90]	Taiwan	Longitudinal	Steatosis and not steatosis	PNPLA3 C > G not associated with cardiovascular‐related death
Lavrado et al. [71]	Brazil	Longitudinal	MASLD and diabetes	PNPLA3 C > G not associated with cardiovascular‐related death
Extrahepatic cancer				
Meffert et al. [85]	Germany	Longitudinal	General population with/without MASLD	PNPLA3 C > G protective against cardiovascular‐related mortality only females without steatosis
Wijarnpreecha et al. [91]	US	Longitudinal	General population with/without MASLD	PNPLA3 C > G not associated with extrahepatic cancer‐related death
Akuta et al. [87]	Japan	Longitudinal	Biopsy‐proven MASLD	PNPLA3 GG associated with higher risk of extrahepatic cancers; data not confirmed at multivariate analysis
Gellert‐Kristensen et al. [81]	Danish	Longitudinal	General population	PNPLA3 C > G not associated with extrahepatic cancer‐related death
Tai et al. [90]	Taiwan	Longitudinal	Steatosis and not steatosis	The impact of FIB‐4 on extrahepatic cancer development evident only in patients with steatosis and PNPLA3 GG
Lavrado et al. [71]	Brazil	Longitudinal	MASLD and diabetes	PNPLA3 CG protective against extrahepatic cancers respect to PNPLA3 CC

Contrasting evidence is available about the impact of PNPLA3 genetic background on cardiovascular risk factors. When looking at the interplay between the *PNPLA3* rs738409 C > G variant and the occurrence of diabetes, in a large Asiatic general population after 4 years of follow‐up the PNPLA3 GG at‐risk genotype disentangles the risk of fatty liver and 2‐h oral glucose tolerance test. Specifically, individuals with the PNPLA3 GG genotype had an increase in steatosis occurrence but a lower rate of development of glycaemic alterations with respect to PNPLA3 CC wild‐type subjects and independently of body weight changes, all these features confirming evidence about lack of effect of PNPLA3 on insulin sensitivity [92]. Along this line, Moon and colleagues demonstrated that in a longitudinal study on a cohort of 8000 individuals who underwent at least 2 health check‐ups, the *PNPLA3* rs738409 C > G variant was associated with a lower risk of developing diabetes in MASLD subjects [HR 0.65], while to a higher risk of occurrence of diabetes in individuals without MASLD [HR 2.4] [93]. These results could suggest that the difference might be explained by the low metabolic risk in patients with genetic PNPLA3‐driven MASLD. Consistent with a protective metabolic effect of the *PNPLA3* rs738409 C > G variant, a recent meta‐analysis on 81 003 subjects from 63 studies showed that the variant was associated with lower triglycerides and total cholesterol serum levels, this effect is more pronounced in Caucasians and obese patients [94]. On the other hand, a Mendelian randomisation analysis reported that genetically determined hepatic steatosis was associated with a small increase in the risk of T2DM in publicly available databases [95]. Despite a potential inverse association between diabetes development and PNPLA3 at risk genotypes, an analysis of 581 individuals with at least 3 metabolic risk factors found a direct and independent association between a procoagulant status assessed by factor VIII/ protein C ratio and both severity of liver fibrosis and the *PNPLA3* rs738409 C > G variant [96]. Consistent with contrasting evidence about the impact of PNPLA3 genotype and metabolic risk factors, there are also heterogeneous data concerning cardiovascular outcomes. A longitudinal study on 4814 participants for the NHANES 1991–1994 cohort reported a trend for an increased risk of cardiovascular mortality in the entire population according to the presence of PNPLA3 G allele, but not in MASLD patients [86]. These data are in line with previous evidence about a higher prevalence of carotid plaques and higher intima‐media thickness in young (< 50 years) biopsy‐proven MASLD patients carrying the *PNPLA3* rs738409 GG genotype [97]. Nevertheless, other studies did not find any effect of the PNPLA3 genotype on cardiovascular events/mortality. This is the case of a Danish population study on 110 913 individuals [83], of a relatively small Asiatic population study [90], of the analysis of 4081 individuals from the Study of Health in Pomerania where a protective effect was shown only in males without steatosis [85], of a Austrian study on 1742 individuals who underwent screening coloscopy [89], of a Swedish study on MASLD patients [51], and of a longitudinal study of 407 patients with diabetes and MASLD [71]. Finally, on the other hand, some studies found an inverse association between the *PNPLA3* rs738408 C > G variant and cardiovascular mortality. A Mendelian randomisation analysis on a large Danish general population cohort confirmed a causal association between liver fat and ischemic heart disease (IHD) but demonstrated that carrying the *PNPLA3* rs738409 C > G variant was associated with a better cardiovascular outcome in terms of IHD [84]. Analysis of data from 48 genome‐wide association studies including 60 801 cases with coronary artery disease (CAD) and 123 504 controls showed that the *PNPLA3* rs738409 G allele was associated with a lower risk of CAD in a recessive adjusted model [98]. Xia and colleagues, in a Chinese population of 5581 adults, observed that subjects carrying the PNPLA3 at risk G allele had a reduced risk of cardiovascular mortality [88]. Along this line, also when moving toward higher‐risk patients, a Japanese study on a cohort of 477 patients with histological diagnosis of MASLD reported that a higher incidence of cardiovascular events was independently predicted not only by the severity of liver fibrosis but also by the presence of PNPLA3 C allele [87].

MASLD and its severity have been also associated with an increased risk of chronic kidney disease [99]. Similarly, growing even if sometimes contrasting evidence suggests a potential role of PNPLA3 genotype on the development of kidney injury. Cross‐sectional studies in a relatively small cohort of patients with diabetes and a large cohort of children reported a direct association between the *PNPLA3* rs738409 C > G variant and lower estimated glomerular filtration rate (eGFR) [100, 101]. However, data on 534 patients with MASLD by ultrasound did not confirm the association between lower kidney function and PNPLA3 C > G variant [102]. When moving to longitudinal studies, in a cohort of 144 patients with metabolic syndrome and with a median follow‐up of 17 months, Mantovani and colleagues observed that the presence of the PNPLA3 G allele was independently associated with more rapid decline in kidney function by eGFR [103]. Similarly, the same group, in a small sample of post‐menopausal females with diabetes, and during a 5‐year follow‐up, observed a faster decline in eGFR in those carrying the PNPLA3 G allele concerning wild‐type patients [104]. Another key topic is the increased risk of extrahepatic cancers in patients with MASLD [105]. In this setting, some contrasting evidence also supports the potential role of genetic background. A large Danish population study on more than 100 000 individuals with a median follow‐up of 9.5 years did not find any association between extrahepatic cancer‐related mortality and PNPLA3 genotype [73]. Similarly, data from a cohort of about 4000 subjects from the Study of Health in Pomerania and with the availability of PNPLA3 genotyping and ultrasound assessment of steatosis, did not report any link between *PNPLA3* rs738408 C > G variant and extrahepatic cancers in the entire cohort, while showing a protective effect (adjusted HR 0.39) only in females without steatosis [84]. Another population‐based study from the NHANES III cohort confirmed the lack of impact of the PNPLA3 G variant on extrahepatic cancer‐related mortality in the entire cohort of 4814 subjects and in the subgroup of 1952 with steatosis [91]. When moving from the general population to patients with steatosis, an Asiatic study on 546 patients with fatty liver found that the PNPLA3 G allele progressively increased the risk of developing extrahepatic cancer even if this association was lost after adjusting for the severity of liver fibrosis [89]. Along this line, a previous quoted Asiatic study on 477 patients with histological diagnosis of MASLD found that the *PNPLA3* rs738409 GG genotype was significantly associated with a 3‐time higher risk of development of extrahepatic cancer, even if this association was lost after adjustment for severity of liver fibrosis [100]. Conversely, a longitudinal study on 407 patients with diabetes and MASLD found a reduced risk of extrahepatic cancers in patients with PNPLA3 CG genotype respect to CC wild‐type, but not in GG homozygous [71].

The evidence of contrasting results about the association between PNPLA3 genotype and extrahepatic complications in MASLD could be related to many key features: (1) All the reported analyses lack a competing risks approach; (2) differences in clinical setting (MASLD vs. general population) and the prevalence of other risk factors could further affect the observed results; (3) lack—in many studies—of assessment of the impact of both liver disease severity and PNPLA3 genotype on extrahepatic outcomes is the variant the causal agent or the PNPLA3‐driven liver damage?; (4) Lack of assessment of other genetic variants that could affect and modulate the risk of extrahepatic outcomes.

## 7 Conclusions

The pandemic of MASLD as the leading cause of hepatic as well as extrahepatic clinical outcomes urgently demands precision medicine approaches and interventions to identify clusters of patients in terms of therapy and follow‐up. In this context, the interaction between ‘traditional’ metabolic risk factors—especially obesity and T2DM—with the PNPLA3 rs738409 C > G variant can account for the observed variation in disease progression and natural contribute to MASLD heterogeneity. Available evidence shows a robust association between PNPLA3 variants and the severity of liver disease and hepatic outcomes; conversely, contrasting evidence is available about the impact of the PNPLA3 G allele on the risk of extrahepatic complications. In conclusion, from the perspective of personalised medicine PNPLA3 genotyping could represent an arrow for better stratification of our patients.

## Conflicts of Interest

The authors declare no conflicts of interest.

## Data Availability

The authors have nothing to report.
